# When enough is enough: Introducing sufficiency corridors to put techno-economism in its place

**DOI:** 10.1007/s13280-024-02027-2

**Published:** 2024-04-26

**Authors:** Richard Bärnthaler

**Affiliations:** University of Leeds, Woodhouse Lane, Leeds, LS2 9JT UK

**Keywords:** Corridors, Housing, Inequality, Sufficiency, Techno-economism, Transformation

## Abstract

Today's ecological crises are entwined with inequality dynamics, yet prevailing techno-economic approaches in climate research and policy fall short in addressing the ecological crisis as distributional crisis. Recognising the limitations of techno-economism, focused on markets (price adjustments) and technology (efficiency gains), this contribution introduces sufficiency corridors as a concept, research field, and policy approach. Sufficiency corridors represent the space between a floor of meeting needs and a ceiling of ungeneralisable excess, i.e. within the sufficiency corridor everyone has enough (to satisfy needs) while no one has too much (to endanger planetary boundaries and need satisfaction). Establishing such corridors entails a process over time that continuously narrows the gap between floors and ceilings, lifting the former and pushing down the latter by strengthening forms of consumption and production that contribute to need satisfaction while shrinking those that do not. The article discusses the profound implications of this approach for how societal reality is reproduced and/or changed, highlighting the need for decisions that eliminate options between and within sectors and in the realms of consumption and production. After addressing questions of decision-making and the potential to realise corridors, the contribution concludes that the growing scientific consensus to *complement* techno-economic approaches with sufficiency measures remains inadequate. Instead, the possibility of a transformation by design hinges on embedding techno-economism within and subordinating it to a sufficiency framework.

## Inequalities and distribution: the missing elements in techno-economist climate research and policy

The richest 1% globally has been responsible for 23% of total emissions growth since 1990 (Chancel 2022). Over the past 25 years, this “polluter elite” (IPCC 2022, 524) has contributed to more than twice as much carbon pollution compared to the three billion people that comprise the poorest half of humanity (Oxfam 2020; see also Gössling and Humpe 2023). Similar inequalities are evident in the (over)consumption of other resources. Savelli et al. (2023) show that the excessive water use by elites exacerbates urban water crises as much as climate change or population growth. Additionally, maintaining economic inequality close to current levels results in a doubling of the energy required to ensure decent living standards for all, allowing all people on Earth to have their basic needs covered (Millward-Hopkins 2022). At the same time, it is now clear that those who suffer most from environmental degradation have contributed the least to it (Chancel 2020).

These selective vignettes underscore a simple but powerful truth: today’s climate and broader ecological crises are, *at their core*, distributional crises, where excess and deprivation, overshoot and shortfall are interconnected (see also Gough 2017; Büchs et al. 2023). Despite these realities, prevailing climate research and policy, deeply entrenched in an ecomodernist, green-growth paradigm, tend to frame the climate crisis as a matter of decoupling resource use and emissions from economic growth. This is envisioned to be achieved through technological advancements (efficiency gains) and adjustments of the price system to correct market failures (internalising “externalities”). However, this techno-economic approach to climate research and policy has not only failed to resolve its own problem definition, as there is no empirical evidence supporting the existence of absolute decoupling anywhere near the speed and scale needed (Parrique et al. 2019; Haberl et al. 2020; Wiedmann et al. 2020; Vogel and Hickel 2023). Efficiency improvements and price adjustments also constitute responses to a problem framing that considers inequality at best an afterthought. As leading climate economist Gernot Wagner proclaims: “It’s tempting to want to stick it to the man. We instead need to stick it to carbon. (…) Inequality is a real (…) problem. But we can’t delay climate action even further for the false hope of solving all the world’s other ills” (cited in Harvey 2023). Such mindsets cannot provide answers to ecological crises *as* distributional crises.

Against this backdrop, this *Perspective* explores sufficiency corridors as a concept, research field, and policy approach aimed at transcending narrow techno-economic mindsets that significantly limit the available range of climate actions. Sect. “Beyond markets and technology: sufficiency corridors” introduces sufficiency corridors, highlighting key distinctions from the prevailing techno-economic paradigm. Sects. “Enabling sufficiency corridors in the realms of consumption and production” and “An inter- and intra-sectoral perspective on sufficiency corridors” delve deeper into the conceptual debate on sufficiency corridors, exploring both production and consumption perspectives as well as an inter- and intra-sectoral view on corridors. To enhance the accessibility of these conceptual reflections, housing—a sector that is both resource- and emission-intensive and crucial for satisfying needs (Coote and Percy 2020; zu Ermgassen 2022)—is discussed for illustrative purposes. Subsequently, Sect. “Three guiding principles for sufficiency corridors: towards a socialist mixed economy” proposes three guiding principles for sufficiency corridors, Sect. “Who decides how much is enough—and how?” addresses questions of decision-making, and Sect. “The potential of realising sufficiency corridors: between utopia and dystopia” dwells on the real-life potentials to realise corridors. Finally, Sect. “Conclusion: putting techno-economism in its place” concludes by reflecting on the role of techno-economic approaches within a sufficiency-oriented climate research and policy paradigm.

## Beyond markets and technology: sufficiency corridors

In the face of escalating inequalities and the mounting evidence that techno-economic approaches cannot deliver the necessary transformation, sufficiency has gained prominence in critical social science climate research (Princen 2005; Max-Neef 2010; Hayden 2019; Jungell-Michelsson and Heikkurinen 2022; Bohnenberger 2023; Gough 2023). Sufficiency revolves around a simple idea: “As one does more and more of an activity, there can be enough and there can be too much” (Princen 2003, 43). The latest IPCC report defines sufficiency as “a set of measures and daily practices that avoid demand for energy, materials, land and water while delivering human wellbeing for all within planetary boundaries” (IPCC 2023, 72). Others describe it as *having enough* in the dual sense of the word, encompassing both a minimum and maximum (Spengler 2016), and as “the space between a floor of meeting needs and a ceiling of ungeneralisable excess” (Bärnthaler and Gough 2023, 1; see also Raworth 2017).

**Fig. 1:**
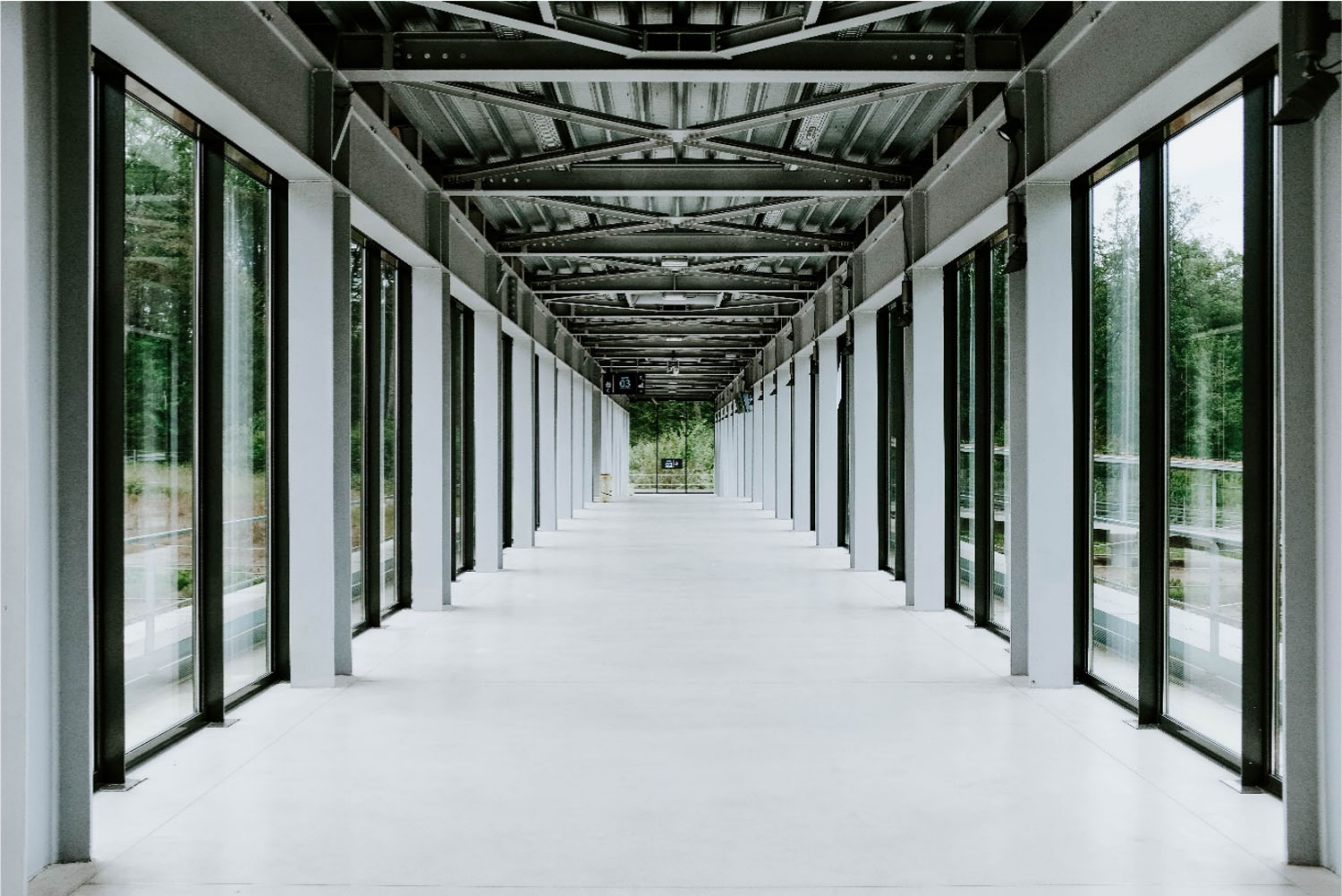
A corridor, source: Freepik.com. Designed by Freepik

“Corridors” are ways to operationalise the principle of sufficiency. In their broadest sense, they establish certain minima, allowing every individual to live a good life, and maxima, ensuring a limit on the use of natural and social resources.^1^ Corridors depict a three-dimensional space and entail a journey through time that continuously narrows the gap between floors and ceilings (see Fig. 1), lifting the former to satisfy everyone’s needs and pushing down the latter to shrink forms of excess that do not contribute to need satisfaction but pursue unlimited wants that endanger planetary boundaries. The continuous convergence of floors and ceilings makes clear how questions of distribution and inequalities are at the core of this concept.

Corridors confront us with a problem framing that constitutes a significant departure from the prevailing techno-economic paradigm. While techno-economism primarily concentrates on means, seeking to maximise the efficient use of resources through technologies and market allocation, corridors shift the focus to ends, i.e. the purpose for which resources are utilised in the first place. These distinctions carry profound implications for how societal reality is reproduced or changed via different modes of human agency, also referred to as “agentic operators”, i.e. “ways of intervening into reality” (Hausknost 2014, 358). Market optimisation enhances *choices* but does not eliminate options—investors and consumers can still choose between more or less sustainable alternatives. Technological advancements refine *solutions* based on the clear criteria of eco-efficiency, aiming to sustain business-as-usual, only more efficiently (ibid., see also Shove 2018).

In contrast, sufficiency corridors involve *decisions* that eliminate options (ungeneralisable excess) in a field marked by “different value systems”—hence, “the *political* and *radical* character of the agentic operator decision” (Hausknost 2014, 361). Rather than further expanding individual choices, the explicit objective is to eliminate unsustainable alternatives, to intentionally unlearn practices of ungeneralisable excess (see also Novy et al. 2022). Going beyond the more efficient reproduction of the status quo, such decisions are inherently value based, involving qualitative shifts. Decisions are not rooted in (aggregated) individual preferences but in collective negotiations about a desired end state within the planetary feasible (see Sect. “Who decides how much is enough—and how?”). The following two sub-sections delve deeper into the conceptual debate on sufficiency corridors.

### Enabling sufficiency corridors in the realms of consumption and production

In recent years, the concept of “consumption corridors” has gained traction to define and operationalise consumption minima and maxima, ensuring certain consumption standards for all while preventing individuals “from consuming in quantities or ways that hurt others’ chances to do the same” (Fuchs et al. 2021, 4). The recent IPCC report underscores the significance of fair demand-side measures and proposes “minimum and maximum standards of consumption or sustainable consumption corridors” (IPCC 2022, 514).

Elsewhere, expanding on this concept, Ian Gough and I (Bärnthaler and Gough 2023) highlight the consumption–production nexus to underscore the *production* of overconsumption. In this context, production decisions are evidently made by those who own and control the means of production and not by final consumers (see “treadmill of production theory,” e.g. Gould et al. 2004). To broaden the focus, we introduce the concept of “production corridors” to complement ongoing discussions on consumption corridors. This extension aims to provide a more comprehensive understanding of climate change as a class struggle, acknowledging that the power over the economy resides with those who own and control production (Malm 2016; Huber 2022).

Embracing both concepts expands the policy space, allowing for corridor-oriented policy portfolios that interconnect consumption and production. The application of this framework is illustrated in the following Table 1, using the example of housing.

**Table 1:** Exemplary policies for consumption and production corridors in the field of housing

	Consumption	Production
Limiting excess	Vacancy taxes and socialisation of vacancies; maximum apartment sizes and progressive land consumption taxes; restricting second homes	Restricting financialised housing production (“not-for-housing housing”)
Guaranteeing social foundations	Housing benefits (subject subsidies)	Providing public housing (object subsidies); introducing zoning categories for affordable housing

### An inter- and intra-sectoral perspective on sufficiency corridors

An additional distinction, not yet explored in the sufficiency-corridor debate, involves differentiating between an inter- and intra-sectoral perspective. *Inter-sectoral corridors* gained prominence during the Covid-19 pandemic, underscoring the varying importance of different sectors. Essential sectors, such as health, care, housing, energy, and water, are vital to satisfy human needs, while others, like the luxury industry and extractive finance, can be suspended without jeopardising human well-being (Gough 2020; Bärnthaler et al. 2021b). Examining sufficiency corridors from an inter-sectoral perspective necessitates such decisions *between* sectors, involving assessments of ‘essentiality.’ As Hoffmann and Spash (2021) recognise, what is ‘essential’ is currently assessed against the aim of reproducing the *present type* of society. This becomes a problem in the pending transformation if sectors classified as essential must “be considerably reduced or even discontinued under climate mitigation agendas, e.g. in the aviation, chemical, steel, or fossil fuel industries, or the defence sector” (ibid., 22). However, inverse cases require decisions too. For instance, if digital platforms are today considered essential, there are compelling arguments for their socialisation—transferring ownership from profit-oriented corporations that extract monopoly rents and providing them as public services of general interest (Bärnthaler et al. 2021a). In navigating these complexities, additional research and democratic debate are imperative to assess, deliberate, and decide on ‘essentiality’ within the context of a social-ecological transformation and to cross-tabulate these decisions against embodied emissions and material throughput.

The *intra-sectoral perspective*, in contrast, involves decisions *within* specific sectors, such as housing or utilities. Recent real-life examples include electricity price controls, where a fixed lower price is established for a basic level of electricity consumption, with consumption above this level priced at market rates. Similarly, interventions addressing water shortages have restricted certain forms of water consumption to ensure basic provision. A notable illustration in the housing sector is the significant inequality in floor space distribution (see e.g. Gough et al. 2024), accompanied by a consistent trend of increasing floor space per capita in most Global North countries (Lorek and Spangenberg 2019, 288).

Against this trend, a global scenario for decent living standards (DLS) with minimum energy suggests 15 m^2^ floor space per capita (60 m^2^ for a four-person household) (Millward-Hopkins et al. 2020), while others, adopting a household perspective, consider 30 m^2^ a sufficient flat size, with an additional 10 m^2^ for each additional household member (Rao and Min 2018). While initially aimed at determining the energy and materials needed for social minima, DLS research is now also employed as an estimate to address social maxima to avoid transgressing planetary boundaries. Although somewhat formulaic, lacking socio-cultural and climatic context, and overlooking specific needs (e.g. disabilities, ageing, non-heteronormative family structures), these estimates can serve as sufficiency benchmarks—to be contextualised within contemporary housing realities.

Especially in the Global North, achieving sufficient floor space demands significant efforts, not only to address associated inequalities but also to reverse the trend of growing floor space per capita, necessitating a fundamental rethinking of housing (see also Durrant et al. 2023): from individual and private spaces to prioritising collective, communal and public ones (“private sufficiency, public luxury”), from the compulsion to possess to the freedom to share (Ivanova and Büchs 2022, 2023), from inflexible housing designs to those adaptable to various stages of life and new living arrangements (Fuhrhop 2020), and from the mandatory creation of parking lots to the obligation to create green spaces (Furchtlehner et al. 2023). These considerations suggest that ‘living space’ is “a more malleable concept than is typically construed to be the case” (Cohen 2021, 181). Inadequate living conditions are not per se a matter of apartment size but often result from deficient collective living environments and inadequate design (see also Novy et al. 2024). These aspects are currently widely overlooked in the ongoing ‘renovation wave’ in Europe, aiming to retrofit millions of homes. Here, overcoming psychological barriers—influenced by societal norms of what constitutes ‘a good life’—associated with reducing one’s private living space and addressing the notable absence of practical alternatives are key challenges (Huebner and Shipworth 2017). Creating such alternatives of redistributed housing space, however, necessitates challenging the prevailing property-rights regime. A revival of the Aristotelian principle of the ‘social obligations of property’ (Szaif 2005; Nuss 2019; Robé 2020), still enshrined in some constitutions but de facto ineffective, would mark a paradigm shift towards tying property more closely to use/needs, fostering more equitable societal and society-nature relationships.

## Three guiding principles for sufficiency corridors: towards a socialist mixed economy

So far, the discussion has centred on addressing minimum and maximum thresholds in the realms of consumption and production, considering both inter- and intra-sectoral perspectives. However, there will always be a space of consumption and production, one that likely needs to shrink over time, that entails more than enough but not too much. In this space, guaranteed consumption and production minima are exceeded without transgressing maxima at a given point in time, i.e. consumption and production fall within the upper and lower limits. The presence of this “in-between” space is applicable not only from an intra-sectoral perspective (e.g. as regards energy and water consumption, floor space, and the like) but also from an inter-sectoral one, where it encompasses sectors that are neither essential nor excessive (e.g. gastronomy, gyms, various household items, entertainment).

This tripartite division provides the foundation for establishing three guiding principles for the development of sufficiency corridors (Bärnthaler and Gough 2023). These principles must be adapted to context, considering factors such as the specific sector, historically evolved provisioning systems, geography, norms, value systems, and, importantly, planetary feasibility, considering biophysical realities:

1. Radically reduce various forms of excess at the top.
2. Allow for regulated^2^ market provision in the in-between.
3. Restrict or replace markets at the bottom via decommodification^3^ to facilitate provision as a social right.

While these guiding principles, effective in the realms of consumption and production, are applicable to both inter- and intra-sectoral approaches, addressing the intra-sectoral “in-between” can be further enhanced through progressive taxation. For instance, with basic floor space requirements secured (principle 3), square metres exceeding this guaranteed minimum should—up until a certain maximum (principle 1)—be provided at market prices, with a progressive land consumption tax reinforcing steering effects (see also Cohen 2019; Jäger et al. 1996). The calculation of such a tax would consider both the active living space of each person and any vacant dwellings (rented or owned) concurrently to address the hoarding of housing. Additionally, space used as garages or parking lots (where potential living or green space is occupied by cars) should be included. From this total amount of square metres (minus the minimum housing space), the tax liability is calculated with a progressive tariff scheme. To enhance its social effectiveness, facilitate various living arrangements, and avoid social hardships, further differentiations of the levy, as well as exemptions, could be stipulated.

In reference to Brie (2021), and in a broad, non-exhaustive sense, I label these three principles as eco-socialist, as they embody a dialectics of communism and liberalism. This dialectic, Brie asserts, is inherent to socialism, which “has always included both the emphasis on individual liberties and the struggle for the commons of a life lived in solidarity” (ibid., 12, own translation). This acknowledgement underscores that safeguarding individual freedom for all requires a struggle against those forces that undermine the social-ecological foundations, from care to nature (Fraser 2022), upon which these freedoms depend. In this context, socialism is viewed as “the form of solidarity between individual claims for freedom and the [de- and uncommodified] communist foundations [of social reproduction] in modern complex societies” (ibid., 17). Consequently, the three guiding principles aim at a socialist mixed economy. They embrace solidarity at the top (eliminating what undermines our social-ecological foundations), communism at the bottom (socialising and decommodifiying the means of reproduction), and liberalism in the in-between (where individuals and businesses may make their consumption and production choices freely and sustainably).

## Who *decides* how much is enough—and how?

Section “Beyond markets and technology: sufficiency corridors” highlighted the pivotal role of the agentic operator *decision* in establishing sufficiency corridors and underscored its distinctions from *choice*, the operator of markets, and *solution*, the operator of technology (see Hausknost 2014). In this context, decisions are invariably value decisions that involve qualitative shifts and are grounded in collective negotiations, not in aggregated individual preferences. Sufficiency corridors prioritise decisions that institutionalise *enough* and thereby introduce a direction of change that is absent from techno-economic approaches, where choices can always be reversed and efficiency lacks an inherent purpose: “One can find efficiencies in harvesting so as to save trees just as well as one can find efficiencies to get every last bit of fibre off an acre of forest land” (Princen 2003, 39). Unlike choices and solutions, decisions regarding lower and upper limits inevitably prompt inquiries about whose ‘limits’ are being represented and who holds a seat at the decision-making table. This underscores that the ecological crisis is not only a crisis of distribution but also of representation and participation—two additional facets of justice.

Establishing limits is inevitably a social, cultural, and political process of collective self-limitation (Kallis 2019; Brand et al. 2021), one that is intricately interwoven with a biophysical reality. But how to overcome the liberal creed of individual free choice and establish instead collective priorities to discern what is crucial for flourishing societies from what is not? How to achieve any form of democratic agreement on floors and ceilings in a capitalist, hyper-marketised and -individualised, high-carbon social formation? While environmental (academic) activists express optimism that democratisation will inherently contribute to a social-ecological transformation, others rightly urge caution. They argue that “the positing of a necessary relationship between green politics and democracy is mistaken, and constitutes an example of wishful thinking on the part of ecological political theorists” (Humphrey 2004, 116; see also Blühdorn 2022). However, despite the absence of a *non-contingent* link between democratisation and limit setting, there exist viable institutional leverage points to facilitate the reconciliation of these two objectives.

Today, a range of experiments in *expert-guided* deliberative or dialogic democracy, such as climate assemblies, are underway (Gough 2022). In these assemblies, a selected sample of lay members, intended to represent the broader population, engages in deliberations and decision-making *based on clear goals*. Aimed to achieve specified climate objectives, these exercises move beyond establishing minimum thresholds; they explore ways of reducing high-carbon consumption and production, effectively lowering the ceiling. Recent assemblies suggest the potential for reaching agreements on sufficiency-oriented policies (Lage et al. 2023), especially when employing a “dual strategy” (Doyal and Gough 1991; Gough 2017). This involves incorporating input from both experts and citizens, fostering a robust science-civic nexus. The dual strategy is crucial for enabling citizens to periodically recalibrate minimum and maximum limits “according to social and ecological developments, new insights, and changing value systems” (Fuchs et al. 2021, 35). It facilitates the democratic development of forms of collective self-limitation *within the bounds* of scientifically informed biophysical realities, guided by approaches like DLS, which also encompass a global justice dimension. Importantly, the outcomes of these deliberations must be translated into general rules, enforced by states as a form of coercion (Bärnthaler 2024; see also Haderer 2023). This necessitates another “double strategy” (Poulantzas 1978). On one hand, it entails a coordinated struggle in civil society, and particularly in the workplace, for economic democratisation, representation, and participation in upstream decision-making. On the other hand, it involves a concerted effort to secure positions within state institutions as public actors (e.g. policymakers) have the mandate to define universally binding rules, which is necessary to “institutionalise mechanisms of restraint” (Princen 2022, 5).

## The potential of realising sufficiency corridors: between utopia and dystopia

Undoubtedly, contemporary growth-driven capitalist political economies structurally oppose sufficiency corridors (Pirgmaier 2020). However, critical conjunctures must be recognised. In recent emergencies, sufficiency strategies have been contemplated and/or implemented, not necessarily driven by conviction but out of sheer necessity. For instance, during the Covid-19 pandemic, critical sectors were prioritised, leading to the shutdown of others. Gas crises prompted many governments to devise plans for potential energy supply cuts to specific industries in the event of a gas shortage. In times of drought and water scarcity, certain forms of excessive water use, such as filling swimming pools or car washing, were prohibited to ensure basic provision.^4^ Furthermore, in the context of current geopolitical shifts, some suggest that framing sufficiency as an issue of international security—aimed at reducing geopolitical dependencies and addressing energy-related risks—could garner political support (Charbonnier 2022). Notably, France has officially declared sufficiency as one of its three pillars towards decarbonisation.

If there is one certainty, it is that transformations—profound changes in various areas of contemporary societies and economies—are inevitable. The real question revolves around whether these changes will occur by disaster or design (Victor 2008), or, as seems most likely, as a combination of both. Whether one invokes Gramsci’s “pessimism of the intellect” (disaster) or “optimism of the will” (design), sufficiency policies emerge as crucial. On one hand, recent examples (as cited above) suggest that having sufficiency strategies ready when disasters strike holds the potential to facilitate just and effective emergency responses. On the other hand, any transformation by design will necessitate mass mobilisation, which, one may argue, demands reconceptualising necessary climate policies as potentially popular social policies.

This brings us back to sufficiency corridors as a concept inherently counteracting social inequalities and benefiting the many (see also Akenji et al. 2021; Bohnenberger 2023). *Having enough* fosters security in uncertain times and is not only a prerequisite in the struggle against deepening ecological crises but also against authoritarian, anti-science, and illiberal movements. Sufficiency corridors empower those who currently lack access to sufficient energy for decent living, who are most exposed to ecological risks but have contributed least to them, who are most detrimentally affected by market-liberal environmental policies like undifferentiated carbon pricing,^5^ and who have had the least say in economic decisions. In short, whether one is an optimist or a pessimist (or both/neither), whether one focuses on ‘doomsday’ warnings or affirmative processes of collective self-limitation (Kallis 2023), there is much to suggest that sufficiency corridors will gain in importance.

## Conclusion: putting techno-economism in its place

This *Perspective* has introduced sufficiency corridors as a concept, research field, and policy approach to address ecological crises *as* crises of distribution, representation, and participation. This problem framing cannot be adequately addressed by market solutions and technological improvements. In contrast, sufficiency corridors pursue the objective of narrowing the gap between lower and upper limits over time to reduce unsustainable inequalities. While techno-economic mindsets rest on *choices* and *solutions* as primary ways to intervene into reality, sufficiency corridors necessitate *decisions* between and within sectors and within the realms of consumption and production, thereby *introducing a direction of change* that is necessary for purposive, planned societal transformations (Hausknost and Haas 2019). The explicit goal is to ensure decent living for all and to eliminate unsustainable options, to intentionally unlearn practices of ungeneralisable excess.

While there is compelling evidence to suggest that sufficiency corridors will become increasingly important, they still hold a subordinate role in climate research and policy. Zell-Ziegler et al. (2021, 2) emphasise that, despite achieving net-zero emissions by 2050 “relying predominantly on technical options of efficiency and consistency” would be “difficult, if not impossible”, sufficiency has not been accorded the status of a genuine field of policy action (see also Gräbner-Radkowitsch et al. 2022). Against this backdrop, there is a growing scientific consensus that techno-economic approaches must be *complemented* by sufficiency measures (IPCC 2022). However, while this insistence represents a positive step, it remains inadequate. Utilising price incentives to discourage unsustainable choices is as important as implementing efficiency measures and expanding renewable energies. However, none of these trends, not even the dynamic growth of renewable energies, has resulted in a substantial decline in the use of fossil fuels or a reduced pressure on planetary boundaries. Efficiency gains and better choices have largely been “eaten up” by additional growth. This development is by no means surprising in a growth economy.

Therefore, as Ulrich (2020, 119, own translation) points out, enhanced market choices and efficiency gains only yield tangible effects when being “embedded within a policy of sufficiency”. He contends that the priority of sufficiency over techno-economic approaches is inherently rational because “nothing is more irrational and uneconomical from a practical point of view than wasting scarce resources and human lifetime, however efficiently, on the realisation of pointless purposes” (ibid, 120). Therefore, sufficiency corridors must not merely *complement* techno-economic approaches; they must take precedence over them. Consequently, the question ‘To what end are resources used?’ *precedes* the question of means, i.e. how these resources are utilised to achieve these ends. Sufficiency-based decisions delimit the space within which choices are made and solutions are sought. In this recalibrated context of subordination, techno-economic approaches, choices and solutions, remain important and, strictly speaking, “only become rational in the first place” (ibid, 115). Taking sufficiency seriously and acknowledging social and biophysical realities in climate research and policy thus necessitates “a different value standard” (Gough 2023), one that puts techno-economism in its place.

## Footnotes

1 Social resources are diverse, ranging from available labour and time to available space.

2 As all markets are regulated (e.g. so-called “free markets” are regulated according to market-liberal rules), regulation in this context refers to *common-good-oriented regulations*. This encompasses measures such as reducing advertising, implementing a right to repair, and enforcing eco-social production standards, including regulations to ensure good working conditions and the production of long-lasting products.

3 Depending on context, these can range from re-nationalisation and -municipalisation to social licensing, commoning, and hybrids like civil-public partnerships.

4 This represents a clear departure from neoclassical economic theory, which views the establishment of collective goals and priorities as unacceptable. In contrast to classical political economy, neoclassical market price theory of value determines demand based on individual consumer preferences, backed by money. As a result, it is considered “objectionable and immoral for government or ‘society’” to declare any economic activity as more or less important than another (Gough 2020). According to this perspective, using water to fill swimming pools is no different from using it to satisfy one’s thirst, and the production of SUVs is deemed equivalent to the production of life-saving ventilators. It is important to note that in practice, neoclassical value theory has undergone modifications during emergencies. Distinctions are made, and collective priorities are set to achieve specific goals, typically within a relatively short period (see Malm 2020).

5 See Oswald et al. (2023) on the fairness-potentials of *differentiated* carbon taxes that distinguish luxury and basic household consumption.
